# Risk factors for renal toxicity after inpatient cisplatin administration

**DOI:** 10.1186/s40360-020-0398-3

**Published:** 2020-03-02

**Authors:** Elena Galfetti, Alessandra Cerutti, Michele Ghielmini, Emanuele Zucca, Luciano Wannesson

**Affiliations:** 1 Kantonsspital Winterthur, Department of Oncology, Winterthur, Switzerland; 2 Biasca, Switzerland; 3 Istituto Oncologico della Svizzera Italiana (IOSI), Oncology Clinic, Via Ospedale 12, 6500 Bellinzona, Switzerland

**Keywords:** Cisplatin, Renal toxicity, Outpatient administration, Hypertension, Cirrhosis

## Abstract

**Background:**

After several decades, cisplatin continues to be an essential drug for the treatment of several tumors, however, its potential nephrotoxicity is still a clinically relevant issue. Identification of predisposing factors for renal toxicity could be of value to warrant prophylactic measures.

**Methods:**

We analyzed data from 198 patients with various tumor types, treated with cisplatin containing regimens in our regional cancer center in a two-years period. Assessed variables included age, gender, smoking status, alcohol consumption, tumor type, prior or concomitant anticancer treatment, cisplatin dose, time-interval between cycles, number of cycles, concomitant nephrotoxic drugs or radiotherapy and co-morbidities. We divided cisplatin nephrotoxicity in two categories: transient and permanent. Univariable and multivariable analyses were performed in order to define statistical associations.

**Results:**

Cisplatin discontinuation rate was 27,7%, of which, 8.1% was due to renal toxicity. A total of 74 and 21 patients developed transient and permanent nephrotoxicity, respectively. At univariable analysis cirrhosis (*p* = 0.027), hypertension (*p* = 0.020), alcohol intake (*p* = 0.030) and number of cycles < 4 (*p* = 0.002) were significantly associated with transient renal toxicity, while at the multivariable analysis, a statistical significance was detected for cirrhosis (*p* = 0.009), hypertension (p = 0.009) and a total number of cycles < 4 (*p* = 0.003). Regarding permanent renal toxicity, a concomitant administration of NSAIDs was significant at univariable analysis (*p* = 0.002).

**Conclusions:**

Relevant risk factors for the development of transient nephrotoxicity were defined. Patients presenting these baseline characteristics may require more frequent post-cycle check-up visits and hydration treatment should be guaranteed as soon as a reduction of creatinine clearance is detected.

## Background

Cisplatin is a potent anticancer drug widely used for the treatment of several malignant tumors. One of the most relevant side effects of cisplatin is nephrotoxicity, that is very well-known since its introduction in 1978 and limits its use and efficacy [1, 2]. Nephrotoxicity prevention has long been managed at the Istituto Oncologico della Svizzera Italiana (IOSI) with hospitalization and forced hydration protocols in order to guarantee a minimum of forty-eight hours of intravenous perfusion. This modality, although associated with a low rate of renal toxicity in our hands, requires considerable resources at the expense of higher costs. For this reason we decided to develop an outpatient cisplatin administration program. Despite the worldwide use of cisplatin for more than 40 years and extensive research on nephrotoxicity mechanisms, we still have limited knowledge about the clinical factors influencing this phenomenon. The purpose of this study is to determine predisposing factors for cisplatin induced renal toxicity in order to allocate our patients with higher risk of renal toxicity to the inpatient strategy and patients at lower risk to the new outpatient cisplatin administration program.

## Methods

We retrospectively analyzed the medical records of all adults patients (*N* = 198) treated with cisplatin-based chemotherapy for various types of tumors at our regional cancer center during the 2 years-period preceding the present analysis. Fourteen patients were excluded from the analysis due to incompleteness of data. In the case of patients treated with more than one cisplatin-containing line, only the earliest line was included in the analysis. We limited the cases to patients who received a dose of cisplatin below 100 mg/m^2^, because for patients who received doses ≥100 mg/m^2^, we decided to continue administering cisplatin upon the hospitalization-based modality. Cisplatin administration protocols complied with the recommendations of the European Society of Clinical Pharmacy published in 2008 [3].

### Selection of variables

Variables potentially associated with nephrotoxicity were selected from previously published studies that investigated renal toxicity induced by cisplatin [4–12].. The following variables were recorded: age, gender, smoking status, alcohol abuse, body mass index (BMI) and tumor type. We also included previous cancer treatments, cisplatin dose per cycle, cumulative dose, type of administration (single day infusion versus dose divided into 2 or more days), time interval between cycles (in weeks), total number of cycles, other cytotoxic drugs administered with cisplatin and concomitant radiotherapy, potentially nephrotoxic drugs received during treatment with cisplatin such as NSAIDs, antibiotics (aminoglycosides, glycopeptides or other class of antibiotics); administration of drugs with nephroprotective potential (diuretics and mannitol); comorbidities (hypertension, diabetes mellitus, vasculopathy, heart disease, cirrhosis and COPD); and laboratory data such as serum albumin and electrolytes. Heart disease, alcohol abuse, cirrhosis and COPD were recorded as dichotomous variables, that is, present or absent, according to the data from the patient’s records, because a more precise sub-classification or grading was not available for all cases.

### Assessment of toxicity

To assess the influence of cisplatin on renal function in the short and long terms, we recorded the baseline creatinine level, the maximum creatinine value a few days after cisplatin administration and the best creatinine value after complete or partial recovery from cisplatin toxicity at variable time points. We calculated the estimated glomerular filtration rate (eGFR) according to the MDRD formula and the creatinine clearance by the Cockroft-Gault formula. We defined renal toxicity as a decrease of ≥25% from baseline values according to both methods. If short-term renal function tests decreased more than 25% from baseline values, the renal toxicity was considered *transient*. Renal toxicity was considered *permanent* if long-term renal function tests remained over the 25% limit.

### Statistical analysis

For the univariable analysis the statistical significance of the selected variables was calculated using Fisher’s exact or chi-squared, as appropriated. Multivariable analysis was performed by stepwise linear regression with *p* < 0.05 for variable-removal and *p* < 0.005 for variable-retention.

## Results

### Patients characteristics

Of the 184 patients, 106 were men and 78 women. Cancer types included head and neck (68 patients, 37%), genitourinary (17 patients, 9.5%), gynecological (23 patients, 13%) pulmonary (28 patients, 15%), gastrointestinal (24 patients, 13%), hematological malignancies, mainly lymphoma and multiple myeloma (14 patients, 7.5%), breast (3 patients, 1.5%), malignant melanoma (3 patients, 1.5%), thymoma (2 patients, 1%), adrenal (1 patient 0.5%) and brain (1 patients, 0.5%). Comorbidities included hypertension (30%), diabetes (5%), heart disease (6%), vasculopathy (10%), COPD (11%), alcohol abuse (15%) and liver cirrhosis (2%) (Table 1).

**Table 1:** Patient’s characteristics

	N of Patients (%)
Sex	
Male	106 (58)
Female	78 (42)
Tumor Type	
Head and neck	68 (37)
Genitourinary	17 (9,5)
Gynecological	23 (13)
Pulmonary	28 (15)
Gastrointestinal	24 (13)
Hematological	14 (7,5)
Breast	3 (1,5)
Malignant melanoma	3 (1,5)
Thymoma	2 (1)
Endocrine	1 (0,5)
CNS	1 (0,5)
Co morbidity	
Hypertension	54 (30)
Diabetes	10 (5)
Cardiopathy	12 (6)
Vasculopathy	19 (10)
COPD	21 (11)
Alcohol abuse	28 (15)
Liver Cirrhosis	4 (2)
Drugs and other agents	
ICM during cisplatin	26 (14)
Metformin	5 (3)
ACE-Inhibitor	13 (7)
NSAID	28 (15)
Aminoglycosides	3 (2)
Glycopeptides	2 (1)
Others antibiotics	36 (19)

Legend: *CNS* central nervous system, *COPD* Chronic obstructive pulmonary disease, *ICM* Iodinated contrast media, *NSAID* Non steroidal anti-inflammatory drug

Twenty-six patients (14%) received iodinated radiological contrast around the date of administration of cisplatin. Five patients (3%) received concomitant treatment with metformin, 13 (7%) angiotensin converting enzyme and 28 (15%) with NSAIDs. In addition, 3 patients received aminoglycoside antibiotics (2%), 2 patients received glycopeptides (1%) and 36 patients were treated with other antibiotics (19%).

For 55 patients (29.9%) cisplatin was not the first-line chemotherapy. Cisplatin was administrated alone (70 patients; 38%), or associated to VP16 (26 patients, 14%), taxanes (9 patients, 5%), other agents (79 patients, 43%) or radiation therapy (79 patients, 42%). Cisplatin was administrated in 1 single day (166 patients, 90%), in 2 consecutive days (6 patients, 3%), in 3 or more consecutive days (12 patients, 7%). The dose range per course was 10 to 100 mg /m^2^, with a mean of 69 mg/m^2^ (SD +/− 25). The range of cumulative doses remained between 40 and 490 mg/m^2^ (mean 219 mg/m^2^, SD +/− 91). Total numbers of cycles ranged from 1 to 8, with a mean 3.32 cycles. A total of 611 cisplatin administrations were recorded in the study population (Table 2).

**Table 2:** Cisplatin-administration characteristics

	N of Patients (%)
Previous chemotherapy	55 (29.9)
Co-Administration	
Cisplatin alone	70 (38)
VP-16	26(14)
Taxanes	9 (5)
Other agents	79 (43)
RT	79 (43)
Days of administration	
1 day	166 (90)
2 days	6 (3)
3 or more	12 (7)
Cisplatin dose per cycle	
Range	10-100 mg/m2
Mean	69 mg/m2
Cumulative dose (Range)	40-490 mg/m2
N of administrations (Mean)	3.32

Legend: *N* Number, *RT* Radiotherapy, VP-16 = Etoposide

### Renal toxicity

Cisplatin treatment was discontinued in 51 cases (27.7%) of which 15 (8.1%) were due to renal toxicity. *Baseline* mean eGFR was 99.69 ml/min (SD +/− 26.61). Mean eGFR was reduced to 79.32 ml/min (SD +/− 30.88) in the *short-term* after cisplatin administration and recovered to a mean of 98.29 ml/min (SD +/− 30.75) in the *long-term* period. Consequently, in the present model, the short-term values represent the *transient* cisplatin nephrotoxicity; while the *long-term* values represent the *permanent* cisplatin toxicity. 74 patients developed transient nephrotoxicity and 21 patient remained with permanent renal function impairment.

The following variables were found to be significantly associated with transient renal toxicity at univariable analysis: cirrhosis (*p* = 0.027), hypertension (*p* = 0.020), alcohol abuse (*p* = 0.030), number of total cycles < 4 (*p* = 0.002) (Table 3). At multivariable analysis, statistical significance was detected for cirrhosis (*p* = 0.009), hypertension (p = 0.009) and a number of cycles < 4 (*p* = 0.003), constituting strong predictors of renal toxicity (Table 4).

**Table 3:** Factors associated to transient renal toxicity at univariable analysis

	N (%)	*p*-value
Sex		
Male	42 (40)	0.91
Female	32 (42)	
Smoke		
No	33 (42)	0.81
Yes	26 (41)	
Former Smokers	12 (36)	
Alcohol abuse		
No	58 (38)	**0.02**
Yes	16 (61)	
Diuretics		
No	36 (34)	0.14
During Cisplatin	32 (48)	
Before or after Cisplatin	6 (54)	
ACE Inhibitor		
No	68 (40)	0.70
Yes	6 (46)	
Metformin		
No	70 (40)	0.07
Yes	4 (80)	
ICM		
No	65 (42)	0.46
Yes	9 (35)	
Obesity		
No	8 (38)	0.07
Yes	6 (75)	
Diabete		
No	68 (40)	0.21
Yes	6 (60)	
Dyslipidemia		
No	63 (40)	0.26
Yes	11 (52)	
Cardiopathy		
No	70 (41)	0.57
Yes	4 (33)	
Vasculopathy		
No	68 (42)	0.37
Yes	6 (32)	
COPD		
No	65 (40)	0.55
Yes	9 (47)	
Hypertension		
No	45 (35)	**0.017**
Yes	29 (54)	
Cirrhosis		
No	70 (40)	**0.015**
Yes	4 (100)	
Hypoalbuminemia		
No	49 (45)	0.40
Yes	10 (55)	
Mannitol		
No	49 (43)	0.92
Yes	23 (42)	
NSAID		
No	58 (38)	0.06
Yes	16 (61)	
Number of cycles < 4		
No	19 (27)	**0.002**
Yes	55 (50)	

Legend: *COPD* Chronic obstructive pulmonary disease, *ICM* Iodinated contrast media, *NSAID* Non steroidal anti-inflammatory drug, *ACE-Inhibitor* Angiotensin converting enzyme inhibitor, *N* Number of patients

**Table 4:** Multivariable analysis of potential nephrotoxic risk factors according to the univariable analysis

	Coef.	95% confidence interval	p-value
Hypertension	0.20	0.05–0.35	**0.009**
Cirrhosis	0.63	0.16–1.10	**0.009**
Number of cycle < 4	−0.21	−0.35 - -0.07	**0.003**

Regarding permanent renal toxicity only concomitant use of NSAIDs was significant at univariable analysis (*p* = 0.002) (Table 5). We were not able to define a multivariable model for late toxicity due to a very low number of events.

**Table 5:** Factors associated to permanent renal toxicity at univariable analysis

	N (%)	p-value
Sex		
Male	13 (13)	0.60
Female	8 (10)	
Smoke		
No	6 (8)	**0.049**
Yes	12 (20)	
Former Smokers	2 (6)	
Alcohol		
No	18 (12)	0.98
Yes	3 (12)	
Diuretic		
No	10 (10)	0.55
During Cisplatin	10 (16)	
Before of after Cisplatin	1 (9)	
ACE-Inhibitor		
No	20 (12)	0.61
Yes	1 (7)	
Metformin		
No	20 (12)	0.58
Yes	1 (20)	
ICM		
No	17 (11)	0.58
Yes	4 (15)	
Obesity		
No	1 (5)	0.57
Yes	1 (12)	
Dyslipidemia		
No	16 (10)	0.08
Yes	5 (23)	
Diabetes		
No	20 (12)	0.83
Yes	1 (10)	
Cardiopathy		
No	21 (13)	0.18
Yes	0 (0)	
Vasculopathy		
No	18 (11)	0.60
Yes	3 (15)	
COPD		
No	18 (11)	0.60
Yes	3 (15)	
Hypertension		
No	12 (10)	0.19
Yes	9 (16)	
Cirrhosis		
No	20 (11)	0.42
Yes	1 (25)	
Hypoalbuminemia		
No	15 (14)	0.70
Yes	3 (18)	
NSAID		
No	13 (8)	**0.002**
Yes	8 (29)	

Legend: *COPD* Chronic obstructive pulmonary disease, *ICM* Iodinated contrast media, *NSAID* Non-steroidal anti-inflammatory drug, *ACE-Inhibitor* Angiotensin converting enzyme inhibitor, *N* Number of patients

## Discussion

The aim of this study was to identify risk factors predisposing to renal toxicity due to cisplatin in order to better select those patients that could be safely treated in an outpatient basis with this cytotoxic drug.

A study conducted by de Jongh and colleagues determined an association of cisplatin renal toxicity and older age, female gender, smoking, hypoalbuminemia, and paclitaxel co-administration [4]. Another study conducted by Anand et al. suggested older age, alcohol intake and renal radiation as significant factors related to nephrotoxicity [5]. Serum albumin, metoclopramide and phenytoin were detected as possible factors affecting renal function at multivariable analysis by Stewart et al. [6]. Daugaard et al. showed that renal toxicity is dose dependent [7, 8, 13].

Other factors associated with significant decrease in eGFR include the frequency of administration, the cumulative dose [10], concomitant use of aminoglycoside antibiotics [11] and other nephrotoxic drugs such as NSAIDs or iodinated contrast [3]. Comorbidities as hypertension, diabetes mellitus and ischemic heart disease also predispose patients to renal function impairment [12]. We found that in our patient population liver cirrhosis, hypertension and a number of cycles < 4 were strong independent predictors for *transient* renal toxicity. However, we were not able to identify independent predictors of *permanent* toxicity, probably due to a low number of patients (12%) who developed a long-term eGFR decrease. It is important to note that a permanent renal function impairment defined as persistent decrease of eGFR > 25% from baseline do not necessarily imply the development of chronic renal failure; this is particularly true for those patients with high eGFR at baseline. Even if predicting permanent cisplatin toxicity appears to be a more relevant objective, predicting transient toxicity may also have a valuable role for example to envisage those patients who may not be able to complete a determinate number of cycles of cisplatin containing adjuvant or curative chemotherapy. For these patients, a safer administration method such as in-hospital cisplatin administration should be preferred. Transient renal toxicity may also increase health care costs and worsen patients’ quality of life. Although we did not perform an analysis to support this, cases developing renal impairment often required supplementary outpatient visits for i.v. hydration or additional hospitalizations.

*Cirrhosis* was not identified as a predisposing factor for cisplatin-induced renal toxicity before our study. Nonetheless, hypoalbuminemia, a frequent finding in patients with liver damage has been previously described as a predisposing factor [4]. In our study hypoalbuminemia was not predictive of toxicity, but it is important to note that data on albumin levels were missing in 30% of our cases. As described above, low albumin levels may lead to higher concentrations of free plasma cisplatin, with the potential of increased toxicity. Intriguingly, cirrhotic patients are frequently treated with spironolactone, a drug that increases cisplatin nephrotoxicity as previously described [14].

*A high number of cisplatin cycles* was previously identified as a risk factor for renal toxicity [15]. In contrast, we found that a number of cisplatin courses equal or lower than 4 was associated with renal toxicity. This could be explained by the fact that cisplatin treatment could have been discontinued early among patients with a worsening renal function, limiting the role of the number of cycles as a patient sorting variable.

We observed that *hypertension* predisposed patients to a transient renal function impairment. To our best knowledge this has not been previously described. Functional renal vascular abnormalities are observed in hypertensive patients [16]. Subclinical kidney damage due to hypertension could constitute a predisposed ground for cisplatin nephrotoxicity.

Strengths of this study include a relatively high number of patients and the large number of variables analyzed.

However, many drawbacks conditioned by the retrospective nature of the study can be pointed-out. On one hand, we could mention the lack of uniformity of the timing for the measurement of creatinine levels, that we selected as the outcome measure. On the other hand, the allocation of comorbidities, such as diabetes, high blood pressure, cirrhosis, etc., which in our study represented some of the independent variables, was based primarily on the information available in the records, that were registered by the treating doctors. Although we sought confirmation of these data from alternative sources, including pathology and laboratory reports or prescription lists, this was not achieved in all cases.

Finally, a more precise grading of the analyzed comorbidities, instead of the assignment of a dichotomous value, such as the characterization of cirrhosis according to the CHILD-Pugh score for example, could have also given further specificity to the results; although, we were unable to collect this information.

## Conclusion

Based on the results of the present study, hypertension and cirrhosis represent risk factors for the development of transient nephrotoxicity in the context of cisplatin-containing chemotherapy treatment. Patients with one or both of these comorbidities may be allowed to receive cisplatin, but more frequent post-therapy planned check-ups of the renal function are recommended. A more aggressive intravenous hydration should be guaranteed whenever a reduction of creatinine clearance of 25% or more is detected from the beginning.

## Data Availability

The datasets generated and analyzed during the current study are available from the corresponding author on reasonable request.

## Abbreviations

ACE: Angiotensin Converting Enzyme
BMI: Body Mass Index
CNS: Central Nervous System
COPD: Chronic Obstructive Pulmonary Disease
eGFR: estimated Glomerular Filtration Rate
ICM: Iodinated Contrast Media
IOSI: Istituto Oncologico della Svizzera Italiana
MDRD: Modification Diet in Renal Disease
N: Number of patients
NSAIDs: Non Steroidal Anti-Inflammatory Drugs
RT: Radiotherapy
SD: Standard Deviation
VP-16: Etoposide
